# Low vitamin D status strongly associated with periodontitis in Puerto Rican adults

**DOI:** 10.1186/s12903-016-0288-7

**Published:** 2016-09-02

**Authors:** Orlando J. Abreu, Dimitris N. Tatakis, Augusto R. Elias-Boneta, Lydia López Del Valle, Rafael Hernandez, Maria S. Pousa, Cristina Palacios

**Affiliations:** 1 Postdoctoral Master of Science in Clinical and Translational Research, University of Puerto Rico, Medical Sciences Campus, PO Box 365067, San Juan, 00936 PR Puerto Rico; 2 College of Dentistry, Division of Periodontology, The Ohio State University, 305 W. 12th Avenue, Columbus, 43210 OH USA; 3 School of Dental Medicine, University of Puerto Rico, Medical Sciences Campus, PO Box 365067, San Juan, 00936 PR Puerto Rico; 4 Graduate School of Public Health, University of Puerto Rico, Medical Sciences Campus, PO Box 365067, San Juan, 00936 PR Puerto Rico; 5 Nutrition Program, Graduate School of Public Health, University of Puerto Rico, Medical Sciences Campus, PO Box 365067, San Juan, 00936 PR Puerto Rico

**Keywords:** Periodontitis, Vitamin D, Hispanic Americans, Puerto Rico

## Abstract

**Background:**

Periodontitis and vitamin D deficiency are both highly prevalent in Puerto Rico. The aim of this pilot study was to evaluate the association between vitamin D levels and periodontal disease in Puerto Rican adults.

**Methods:**

A sex-, age-, and BMI-matched case-control, cross-sectional study was conducted on 24 cases of moderate/severe periodontitis and 24 periodontally healthy controls aged 35 to 64 years. Each participant completed a socio-demographic questionnaire, underwent a full-mouth periodontal examination and provided blood sample to measure serum 25-hydroxyvitamin D (25 (OH) D) levels to assess vitamin D status.

**Results:**

A total of 19 matched case-control pairs (28 females, 10 males) completed the study. Mean serum 25 (OH) D levels were significantly lower in cases (18.5 ± 4.6 ng/ml) than in controls (24.2 ± 7.1 ng/ml; *p* = 0.006). Lower odds of periodontal disease were observed per unit of 25 (OH) D level (OR 0.885; 95 % CI 0.785, 0.997; *p* < 0.05).

**Conclusions:**

Lower serum vitamin D levels are significantly associated with periodontitis in Puerto Rican adults.

## Background

Periodontitis is an inflammatory disease of the supporting tissues of the teeth that is caused by specific microorganisms and results in the progressive destruction of the periodontium through the elicited inflammatory host response [1]. There is a considerable global variation in periodontal disease prevalence, although the prevalence of severe periodontitis is higher in developing countries [2]. A study reported by the Centers for Disease Control and Prevention (CDC) in 2012 indicated that nearly half of US adults (47 %) have periodontitis [3]. When the prevalence of moderate/severe periodontitis was compared between US and Puerto Rican populations, a significantly higher prevalence was found in Puerto Rican adults (44.5 %) relative to data in adults from National Health and Nutrition Examination Survey (NHANES) 1999–2004 (20.7 %) [4].

Serum vitamin D levels play an important role in oral homeostasis, and dysfunctions in vitamin D metabolism are associated with periodontal disease [5–7]. An adequate serum vitamin D level, as measured by 25-hydroxyvitamin D (25 (OH) D), is considered to be ≥20 ng/ml, according to the Institute of Medicine [8]. However, the Endocrine Society has suggested that >30 ng/ml may be the optimal level for overall health [9]. In fact, optimal vitamin D levels have shown additional benefits to oral health [10, 11] but there are limited studies on the relationship between vitamin D and periodontal status.

Vitamin D deficiency is a worldwide public health concern [12]. About 32 % of the US population may have vitamin D deficiency (<20 ng/mL) [13], which increases during the winter [14]. Hispanics are at a higher risk of vitamin D deficiency than are non-Hispanic whites [15–18]. Puerto Ricans in particular are among the Hispanic groups with the highest vitamin D deficiency prevalence, as evidenced by a study of 358 Hispanic-American men, which found that those born in Puerto Rico displayed the highest prevalence of vitamin D deficiency (26 %) compared with participants from the Dominican Republic (21 %), Central America (11 %), and South America (9 %) [19]. A recent report from a large sample of 2,293 Puerto Rican adults demonstrated that 28 % had vitamin D deficiency and that 72 % had vitamin D insufficiency (levels <30 ng/ml) [20]. Furthermore, we recently reported that that 77 % of participants in a Puerto Rico endocrinology clinic had low vitamin D status (levels <30 ng/ml), while only 23 % had optimal levels (≥30 ng/ml) [21].

Studies investigating the possible association between periodontal disease and vitamin D status in Hispanic populations, in general, and Puerto Ricans, in particular, are lacking. The high prevalence of both periodontitis and low vitamin D status among Puerto Ricans suggested an important need to address this knowledge gap. Therefore, the present study aimed to examine whether an association exists between vitamin D status and periodontal disease in Hispanic adults living in Puerto Rico. We hypothesized a lower periodontal risk as serum 25OHD levels increased.

## Methods

### Study design

A matched case-control study was conducted in 48 adults (24 cases with moderate/severe periodontitis and 24 periodontally healthy controls). Cases and controls were matched for sex, age (±10 years), and body mass index (BMI; ±2 kg/m^2^). Cases were recruited first and then controls were recruited to match these covariates. This study was approved by the Institutional Review Board of the University of Puerto Rico, Medical Sciences Campus (UPR-MSC). Participants signed a consent form before participating in the study.

### Study population

Male and female Hispanic adults aged 35 to 64 years were recruited from external clinics of the School of Dental Medicine at the UPR-MSC. Participants completed a consent form, and questions and concerns were answered and discussed before the study began. Exclusion criteria included individuals with less than 14 teeth at the time of evaluation (necessary to have a proper and meaningful assessment of the participants’ periodontal condition) [22–25], diabetes mellitus type 1, uncontrolled diabetes mellitus type 2 (defined as not checking glucose levels periodically and not under treatment with their primary physician), osteoporosis, prophylactic antibiotic therapy needed before dental procedures (e.g., prosthetic cardiac valve, history of infective endocarditis, congenital heart disease), pregnancy, smokers, participants currently undergoing orthodontic therapy and individuals unable to read, understand and sign the informed consent form.

### Measures

Each participant completed a questionnaire to provide information on socio-demographics. Then, participants underwent a full-mouth periodontal examination, and a venipuncture blood sample was collected.

#### Socio-demographics

The questionnaire included self-reported age, sex, weight and height. BMI was calculated by dividing weight (converted to kg) by the squared height (converted to m).

#### Periodontal examination

A periodontal exam was performed by a National Institute of Dental and Craniofacial Research (NIDCR)-calibrated dentist. The examination included recording of probing depth (PD) and attachment loss (AL) at 6 sites (mesiobuccal, buccal, distobuccal, distolingual, lingual and mesiolingual) on all teeth, excluding third molars. Cases were classified as moderate or severe periodontitis based on the 2003 definitions by the CDC and the American Academy of Periodontology Working Group for moderate periodontitis (≥2 interproximal sites with AL ≥4 mm (not on same tooth) or ≥2 interproximal sites with PD ≥5 mm (not on same tooth); and severe periodontitis (≥2 interproximal sites with AL ≥6 mm (not on same tooth) and ≥1 interproximal sites with PD ≥5 mm) [26]. Controls were periodontally healthy (with neither moderate nor severe periodontitis at the time of examination).

#### Vitamin D status

A 5-ml blood sample was collected by venipuncture by a trained phlebotomist for the measurement of serum 25 (OH) D levels. Serum separator tubes were used, and serum 25 (OH) D levels were measured using a commercially available direct competitive chemiluminescence immunoassay (Liaison, DiaSorin S.p.A., Saluggia, VC, Italy). For this study, vitamin D levels were categorized as deficient if levels were ≤12 ng/ml, inadequate if levels were 12–19 ng/ml, and adequate if levels were ≥20 ng/ml, as suggested by the Institute of Medicine criteria [8]. However, we further divided the adequate group into 20–30 ng/ml (adequate) and >30 ng/ml (optimal) as suggested by the Endocrine Society for optimal health [9]. Although there is little variation in UVB irradiation levels associated with the month in Puerto Rico, the study was conducted during the months of May, June and July 2014 to decrease seasonal variability.

### Statistical analysis

Descriptive statistics included measures of central tendency with the associated variability (e.g. mean and standard deviation) for continuous variables and frequencies and percentages for categorical variables. Normality was assessed using the Shapiro-Wilk test. To compare means between groups, Student’s *t*-tests were performed for continuous variables. The Q de Cochrane test was performed to compare the three categories of vitamin D status (inadequate, adequate and optimal) between cases and controls. To assess the association between the outcome (periodontal disease) and the exposure variable (serum 25 (OH) D levels as a continuous variable), a multivariate matched analysis using a conditional logistic regression model was used. Data analyses were performed using the STATA version 13 software (StataCorp LP. College Station, TX). The significance level was set at *p* < 0.05.

## Results

A total of 19 cases and 19 controls matched for age, sex and BMI completed the study. Table 1 shows the study population characteristics. Among the cases, 9 had moderate periodontal disease, and 10 had severe periodontal disease. Mean AL and PD were significantly higher in the cases relative to the controls (*p* < 0.05). In contrast, mean serum 25 (OH) D levels were significantly higher in the controls relative to the cases (*p* < 0.05).

**Table 1:** Study sample characteristics (mean ± SD or N (%))

Variable	Cases (*n* = 19)	Controls (*n* = 19)
Age (y)	47.6 ± 8.7	46.7 ± 8.2
Sex (females/males)	14 (73.7 %)/5 (26.3 %)	14 (73.7 %)/5 (26.3 %)
BMI (kg/m^2^)	29.5 ± 5.1	29.3 ± 6.0
PD (mm)	2.3 ± 0.4	1.3 ± 0.3^a^
AL (mm)	2.6 ± 0.6	1.3 ± 0.4^a^
Serum 25 (OH) D (ng/ml)	18.5 ± 4.6	24.2 ± 7.1^b^

^a^Significantly different from cases (*p* < 0.0001; Student’s *t*-test)

^b^Significantly different from cases (*p* = 0.006; Student’s *t*-test)

*BMI* Body mass index, *PD* probing depth, *AL* attachment level

Table 2 shows vitamin D status in the two groups using the Institute of Medicine and the Endocrine Society cutoff points. Serum 25 (OH) D levels were normally distributed in each group. No participant had vitamin D deficiency, but we observed a greater proportion of controls with optimal levels of 25 (OH) D relative to cases and a concomitantly greater proportion of cases with inadequate levels of 25 (OH) D (*p* < 0.05). The analysis of the conditional multiple logistic regression showed that for every unit (ng/ml) increase in serum 25 (OH) D levels, the odds of periodontal disease was significantly reduced by 12 % (OR 0.885; 95 % CI 0.785, 0.997; *p* < 0.05).

**Table 2:** Vitamin D status according to periodontal status (periodontitis cases and controls)

Vitamin D status categories	Cases		Controls		Q Cochrane
	*N*	%	*N*	%	*P* value
Deficient ≤12 ng/ml	0	0.0	0	0.0	<0.001
Inadequate (25OHD = 12-19 ng/ml)	12	63.2	6	31.6	
Adequate (25OHD = 20-30 ng/ml)	7	36.8	9	47.4	
Optimal (25OHD > 30 ng/ml)	0	0.0	4	21.1	

## Discussion

In the present pilot case-control study in a sample of Puerto Rican adults, periodontitis cases had significantly lower levels of serum 25 (OH) D compared to controls; furthermore, for every unit increase in serum 25 (OH) D levels, the odds of severe and moderate periodontitis was significantly reduced by 12 %. To our knowledge, this is the first study to evaluate the relationship between serum 25 (OH) D levels and periodontal disease in a group of Hispanics. The periodontal status of the cases recruited in this study appears representative of the Puerto Rican population at large, as reported in epidemiological studies [4]. Similarly, the vitamin D status of the controls recruited in the present study matches the previously reported status of much larger Puerto Rican adult population samples [19–21].

Other studies have also found an association between vitamin D status and periodontal disease. In a case-control study on pregnant women, involving 117 cases (clinically moderate to severe periodontitis) and 118 controls (periodontally healthy), cases presented with lower median 25 (OH) D levels (23.6 ng/ml) relative to controls (40 ng/ml; *p* < 0.001) [27]. Pregnant women with periodontal disease were more likely to have serum 25 (OH) D levels <30 ng/ml (65 % of cases versus 29 % of controls; *p* < 0.001); furthermore, pregnant women with 25 (OH) D levels <30 ng/ml had a twofold increase in the odds of moderate to severe periodontal disease (adjusted OR 2.1; 95 % CI 0.99, 4.5). Similarly, a prospective study of 42,730 adults aged 40–75 years participating in the Health Professionals Follow-Up Study and followed for 20 years found that participants in the highest quintile of the predicted 25 (OH) D score had a 20 % lower incidence of tooth loss compared with the lowest quintile of 25 (OH) D score (HR 0.80, 95 % CI 0.76, 0.85; *P* < 0.001) [28]. However, a case-control study of Finnish adults, with 55 cases (chronic periodontitis) and 30 controls (periodontally healthy), did not find an association between 25 (OH) D levels and periodontal health status [29]. This latter study found that participants with low 1,25 (OH) 2D were more likely to be in the case group (OR = 0.97, 95 % CI = 0.95-1.00). The lack of association between 25 (OH) D levels and periodontal disease in the Finnish study was attributed in part to the overall lower serum 25 (OH) D levels among study subjects.

Vitamin D insufficiency plays a role in dental (altered formation) and oral bone pathologies (altered formation, periodontal disease and jaw osteonecrosis), the mechanisms of which rely on the specific behavior of oral cells and dental cells, which are responsive to vitamin D [30]. Oral epithelial cells are capable of converting inactive vitamin D to the active form of 25 (OH) D, which has been shown to induce expression of the antimicrobial peptide LL-37 and other host defense mediators [31]. This may represent a mechanism by which vitamin D enhances innate immune defenses against periodontal pathogenic bacteria. In fact, another study found that optimal ranges of serum vitamin D may reduce susceptibility to gingival inflammation and that gingivitis may be a useful clinical model to evaluate the anti-inflammatory effects of vitamin D [5]. This finding was supported by the results of a more recent randomized clinical trial, which showed that vitamin D has a dose-dependent anti-inflammatory effect on gingivitis [32]. Furthermore, vitamin D may also reduce periodontal disease through its general anti-inflammatory and immunomodulatory effects [33, 34].

Study limitations and strengths should be taken into consideration when interpreting the reported results. Sample size and lack of inclusion of other confounders (e.g., socio-economic status and physical activity) are two of the study limitations. Nevertheless, all participants were recruited from the university external outpatient clinics, a service provided at a lower cost to the community, thereby ensuring a homogenous sample with similar socio-economic status. In terms of study strengths, the same selection process was used to recruit both cases and controls, while periodontal status of all participants was assessed by a NIDCR-calibrated dentist. In addition, we used serum 25 (OH) D levels to assess vitamin D status, which reflects both dietary intake and sun exposure, and these were measured in the same laboratory with identical methodology.

## Conclusions

In conclusion, lower serum vitamin D levels are significantly associated with periodontitis in Puerto Rican adults. These results suggest that there is value in screening periodontitis patients for low vitamin D status. Island-wide studies are needed to confirm these findings and analyze additional determinants of periodontal health in Puerto Ricans.

## Abbreviations

25 (OH) D: Serum 25-hydroxyvitamin D
AL: Attachment loss
BMI: Body mass index
CDC: Centers for Disease Control and Prevention
HR: Hazard ratio
NHANES: National Health and Nutrition Examination Survey
OR: Odds ratio
PD: Probing dept
UPR-MSC: University of Puerto Rico, Medical Sciences Campus
UVB: Ultraviolet B
